# Adolescent Neurological Development and Implications for Health and Well-Being

**DOI:** 10.3390/healthcare5040062

**Published:** 2017-09-29

**Authors:** Angela Griffin

**Affiliations:** Child Psychology Department, Southampton Children’s Hospital, Mailpoint 133, Block 8, Tremona Rd., Southampton SO16 6YD, UK; angela.griffin@uhs.nhs.uk

**Keywords:** adolescence, brain development, prefrontal cortex, neurological development, chronic health condition

## Abstract

Adolescence is evolution’s solution to bringing the capacity of our large, complex brains to fruition. It is a critical period for brain development and the experiences of each adolescent during this time helps to shape their adult brain. Brain developments lead to both the hormonal changes and the emotional, cognitive, and behavioral characteristics of the teenage years. They drive a growth towards independence via more complex reasoning skills, increased importance of social affiliations outside the family, and an urge to experiment and explore boundaries. In the context of still incomplete inhibitory systems, a heightened sensitivity to rewards, including the need for social acceptance, can mean risk-taking or impulsive behaviour in some. The continued plasticity of the brain can also mean a creativity and openness to novel solutions. These normative steps of adolescence are especially relevant to young people with chronic health conditions. An understanding of brain development at this time can help us appreciate the perspective and priorities of adolescents with health conditions. It can also guide us towards better ways of collaborating with them.

## 1. Brain Development in Adolescence

The brain’s growth has largely been done before adolescence but it undergoes extensive remodelling in the adolescent years. The focus is no longer on the proliferation of neurons, but on strengthening the network of connections between them. Brain imaging done since the 1990s has shown that brain development progresses in a wave from areas at the back that look after older and more basic functions, such as vision, movement and processing, to the evolutionarily newer and more complex thinking areas at the front of the brain. Much research remains to be done if we are to have a full understanding of this complex and creative time for the brain, but the advent of new technologies, such as diffusion tensor imaging, means that our knowledge is set to increase dramatically in the near future.

We know that children’s brains process information in a less efficient, location-specific way and that one of the key differences that come with maturation, is that processing occurs in a more efficient, connected, and integrated way. Cognitive processing becomes more distributed. Long range connections increase, allowing integration between distant brain regions. This leads to increased processing resources and greater efficiency.

The brain’s wiring changes and develops in a number of ways. The brain’s axons gradually become more insulated by myelin (which gives ‘white matter’ its appearance). Myelin changes are dramatic during adolescence, with the amount of myelin doubling in some brain regions. The insulating effect means impulses or messages can pass at far higher speed along the axon. At the same time, dendrites, which receive transmissions from nearby axons, grow more branches, increasing their connectivity.

The synapses, the chemical junctions at which messages are passed on, grow stronger if they are used often. Those less used, gradually diminish. This is known as synaptic pruning and it causes the brain’s cortex—the outer layer of ‘grey matter’ which does much of our complex, conscious thinking—to become thinner and more efficient. This capacity for plasticity means that experience plays an important role in determining which synapses are eliminated and which are strengthened and reinforced.

Meanwhile, the corpus callosum which bridges the two hemispheres, thickens, improving the efficiency of communication both between and within the brain’s hemispheres.

Stronger links develop between the prefrontal cortex and other brain regions. For example, connections between the hippocampus and frontal areas are strengthened, enabling adolescents to become gradually better at integrating memory and experience into their decision making.

Many of the same structures are involved in emotional processing, in evaluating risk and in controlling impulsivity. These include the hippocampus, the amygdala, the nucleus accumbens and the prefrontal cortex, all of which undergo change during adolescence.

The nucleus accumbens in the ventral striatum is heavily involved in reward processing and evaluation and it is more active in adolescents than in children or adults [1]. It provides the drive for goal-directed behaviour by linking the emotion centres with memory and with the movement system.

The amygdala’s role in fear processing is well-established and this is related to how risk is assessed. The hippocampus has a key role in encoding emotional memory via its connections with the amygdala. There is evidence that pubertal status and gender affects their sizes, but the nature and direction of their influence is still unclear [2].

The defining feature of our human brain is our late-evolving pre-frontal cortex, the first third of the frontal lobes. One of the key goals of the pre-frontal cortex is to become skilled at reconciling internal emotional states with the demands of external reality.

While the deeper subcortical areas have done most of their development by adolescence, the prefrontal cortex enters a critical period. It is now thought that it may not fully mature until the mid-twenties or even later. It is central to the development of theory of mind, decision-making, and the regulation of complex social behaviours [3,4].

As the pre-frontal cortex develops, utilising feedback from the environment to shape its progress, we learn how to manage long term planning, monitoring what is going on and adjusting smoothly while keeping our emotions and behaviours appropriate to the context [5]. This ability is assisted by the growing inhibitory influence of the prefrontal dopamine system, which gradually improves the ability of teens to ‘apply the brakes’.

Dopamine, and other neurotransmitters, are considered to be a more important influence than hormones in the regulation of emotions, cognition, and behaviour. Changes in neurotransmitter systems accelerate during adolescence, although our knowledge of them is still limited. The dopamine system has been most closely studied. As well as having an inhibitory effect in the prefrontal cortex, it has also been found to be involved in the brain’s response to novelty and rewards in the environment as well as risk-taking behaviour.

Brain regions and networks involved in the perception of facial emotions, taking other people’s perspective, and empathic responding are also not fully mature until adulthood and social behaviour is still a skill under construction.

### 1.1. Sex Differences

The brains of adolescent boys are typically larger in volume and with a higher ration of white matter. Girls have been shown to have thicker cortical volumes in the posterior temporal and inferior parietal areas, even after corrections for brain and body size [6].

However, bigger does not necessarily mean better when it comes to brain size and nor does smaller always mean more efficient. Different brains may do things differently, as shown by the finding that males and females can show different patterns of neural activation while performing a task, but do it equally well [7].

### 1.2. The Interaction between the Adolescent and Their Environment Is Key

While myelin speeds up communication within the brain, it also limits the brain’s ability to rapidly adapt its networks to fit with changes in environmental circumstances. White matter changes are thought to be strongly influenced by experience, so adolescence is a particularly significant time.

The adolescent’s experiences also play a critical role in determining which synapses are heavily used and which are gradually abandoned. As the less used pathways and connections are eliminated, processing becomes more efficient but more limited as the range of processing options are reduced. This highlights the importance of the environment and the experiences adolescents are exposed to in moulding the brain. It also shows that adolescent brains are balanced between having a capacity for being highly adaptive, flexible, and creative alongside a gradual reduction in this as they gain increased efficiency and processing speed.

## 2. What Brain Developments Look Like in the Day to Day Life of Teenagers

The gradual brain developments which take place during the adolescent period show themselves in behavioural, cognitive, and emotional changes. This critical period for brain development enables adolescents to negotiate their trial and error journey towards independence.

### 2.1. Cognition

The maturation of the prefrontal cortex, as well as the improvements in connectivity and changes in neurotransmission that occur, allow an increase in intellectual capacity and in abstract reasoning skills.

Adolescents become increasingly sophisticated in their processing of new knowledge and gradually move from a ‘right or wrong’ framework to a growing tolerance for ambiguity and uncertainty.

In many cases, decision making improves in a linear fashion [8]. In some however, it is thought to follow a U-shaped pattern, improving in childhood, with gains slowing in adolescence before resuming progress in adulthood [9].

Although decision making is a cognitive process, it is highly influenced by emotion. We know adolescents are not as able as adults to inhibit emotional responses. We also know they process rewards, an emotional process, differently to adults and in a way which may drive risk taking, especially if those rewards relate to acceptance by peers [10,11].

### 2.2. Friends over Family

During adolescence connections with peers are valued as never before and they develop via friendships, romantic relationships, and affiliations with a teenage ‘tribe’ (e.g., the sporty group, the ‘geeks’, the ‘non-conformists’, the ‘academics’) [12]. Emotional support from friends can exceed that from family members [13].

As adolescents become concerned with fitting in, they become more aware of how others see them, increasing their sense of self consciousness [14] (Somerville et al., 2013). They are more concerned with their appearance and body image, particularly in terms of how they are evaluated by peers and by potential romantic partners.

Today’s teenagers have to manage this increased sensitivity to judgment and desperation for peer acceptance in a world of social media and selfies!

Some young people are rejected or ignored by peers, or even worse, actively victimised. Moor et al., (2012) identified a network of brain regions involved in the experience of social rejection which includes parts of the prefrontal cortex, the anterior cingulate cortex and the insula [15]. Young teens show greater sensitivity to the negative effects of this than older teens. The existence of protective factors such as family support, having one good friend, or having a particular talent, can modify the impact, but social exclusion may lead some young people to show more risk taking in order to win peer approval. Others may be more at risk of low mood or social anxiety [16,17].

Peer victimisation, both overt and covert (e.g., cyber), has a huge impact on mental and physical health. There is a direct link between cyber victimisation and increased sleep problems and reporting of somatic problems. It causes a decline in self-esteem and an increase in feelings of anxiety, low mood, and loneliness [18].

### 2.3. Emotion Regulation, Experimenting, and Exploration

Emotion regulation is a major influence on behaviour. As mentioned earlier, the prefrontal cortex as well as the striatum region is important in emotion processing, risk-taking, and impulsivity. Reward and threat sensitivity are heavily involved in emotion regulation and are used to study emotion regulation in adolescence.

We typically think of adolescents as having an urge to experiment and explore. Many, but not all, are drawn to temptations, such as testing their alcohol limits, seeing how fast their car can go, or how high a wall they can jump from. It is not so much that they think they are bullet-proof as that the lure of some rewards are so great, the risk seems worth taking. Pubertal status (not chronological age) is linked to a heightened sensitivity to rewards compared to childhood and adulthood [19]. This has a direct impact on risk taking behaviours. The most desirable of rewards at this time is often social status, so high risks are taken with the goal of impressing peers [20]. Teenagers take more risks if they know their peers are watching [21].

Of course sometimes risky behaviour can also result in positive gains, for example, in taking the risk of asking someone out on a date on the slimmest of hopes [5].

Importantly, adolescence is also thought to involve a greater sensitivity to the effects of stress, which has implications for how young people manage to deal with the increasing demands placed upon them and other stressors in their environment, e.g., family conflict. This sensitivity can be further heightened in young people who have experienced chronic stressors, such as abuse or trauma, in childhood. The development of neuroregulatory systems is affected (e.g., the hypothalamic-pituitary-adrenal axis), making it harder for young people to exercise cognitive control and to regulate their emotions and behaviour. Thus, difficult situations, such as peer rejection, can be harder for them to manage. Secure attachment relationships early in life build the brain’s capacity to self-regulate behaviourally and emotionally, ameliorating the impact of stress. 

Higher levels of social competence provide a protective element in the face of social exclusion or peer rejection. While these adolescents may experience the pain of rejection just as intensely, fMRI studies show greater activation in the right ventrolateral prefrontal cortex and ventral striatum during exclusion. This is thought to indicate that they are better at regulating their distress [22].

### 2.4. Sleep

As a result of usual brain development, sleep onset starts to occur later than in childhood. This ‘phase shift’ means many teens, but not all, move to an evening chronotype. It is thought that the adolescent sleep–wake cycle is affected by the locus coeruleus-norepinephrine system which is considered to be the centre of homeostatic regulation and the suprachiasmatic nucleus of the hypothalamus, the brain’s timekeeper.

The environment can push sleep onset later again, due to lighting, television, mobile phones, and social media. Getting to sleep will be even harder if teenagers are exposed to cyber bullying which may occur late at night. As the school day still starts early, this can mean sleep deprivation for many. Research shows that, as a group, teenagers are more tired at 10 a.m. than they are at 10 p.m. [23].

This changing circadian rhythm is a likely contributor to some of the ‘typical’ characteristics of adolescents. They can still be tired in the morning and grumpy as a result. They tend to sleep in late on the weekends as their brain and body tries to catch up on sleep missed during the week.

## 3. Implications for Health and Well-Being

If fitting in is a priority for most adolescents, what does that mean for the teenager with a chronic health condition? Physical symptoms such as pain and fatigue, combined with a need for strict disease management regimes, inevitably interfere with many aspects of daily adolescent life, such as attending school and keeping friendships going [24].

Problems with peers are related to disease control. Friends can either hinder or help young people with chronic health conditions to manage.

If there is family adversity, friends can be a strong protective factor. They can provide companionship in the lifestyle required by the illness, for example, doing exercise together or helping people to stay on track in eating the right foods. Close friends are also often the first to notice if help is needed, and provide reminders to check blood glucose or take insulin. Strong friend support is not always found to be related to adherence to treatment but it is found to be positively related to quality of life [25].

Sometimes strong connections with friends can decrease self-care and it is hypothesised that this is related to the young person’s attempts to mask the need for a specific regimen of care, in order to fit in.

Chronic illness is a risk factor for psychological challenges such as depressive symptoms [26]. Children and adolescents with chronic conditions are somewhat lonelier than their peers without such conditions [27]. Young people with neurological disorders or obesity are found to show less social competence than their well peers [28]. Those with visible differences are more likely to be rejected or victimised by their peers [18,29]. Some young people go unnoticed by their peers. This is more likely if they are young people who are missing from school a lot due to illness or hospital stays and if they are less able to participate in activities when they are there.

Many adolescents enter into romantic relationships which can be significant for their health management. When romantic relationships are caring and supportive, they can increase self-worth and social competence and have a positive effect on disease management. However, Helgeson (2015) reports that in a group of young people with diabetes, romantic conflict was associated with poorer self-care [25].

The groups young people affiliate with can have implications for health [12]. The ‘non-conformists’ show the least healthy patterns, with greater drug and alcohol use. While the sporty group are high on exercise they also tend to consume more alcohol than average. The ‘popular’ group, particularly the girls, tend to drink alcohol and may also have weight control issues. The healthiest group tend to be the ‘brainy/academic’ group.

### 3.1. Promoting Psychological Well-Being: Learning Points for the MDT

Disease management is psychologically mediated. What matters to young people psychologically can be very different to what matters medically. How young people feel about their condition, whether they feel it is acceptable to their peers, or whether they are rejected or ignored socially, are all factors which can impact on disease management and health.

**Figure 1:**
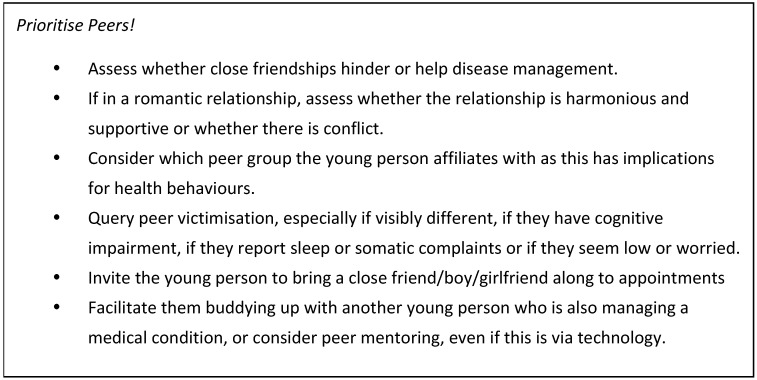
Assessing and utilizing peer relationships.

Given the paramount importance of their social context, those of us supporting young people need to explore this and utilise it (see Figure 1). Finding the best ways to build social competence may mean that adolescents do not need to take risks in order to win friends and are better placed to choose helpful friendships and peer affiliations.

Psychoeducation on the safe and appropriate use of social media can facilitate appropriate online peer relationship skills and may reduce future peer victimisation experiences. Keeping pace with developments in technology is a challenge and interventions may need to be regularly updated and adapted.

It may be challenging for young people who have not had the experience of secure attachment in childhood to engage with the help we are offering. Taking time to slowly build a trusting relationship at their pace may pay dividends in the long run, even if it feels like this initially delays getting the real ‘work’ done.

Although we offer help with the best intentions, we may be more useful if we ask young people what they would like and how they want it to look. For example, in a study of young people with cystic fibrosis, 85% of clinicians said they discussed body image in clinic, but 75% of young people said they had not! Young people said they preferred to discuss body image separately from physical health, whereas providers tended to talk about it along with weight and health issues [30].

One advantage of a brain that is still being refined is that adolescents are better than adults at novel problem solving. Make space to invite their solutions to managing the dilemmas they face in keeping well. Solutions that are constructed together have more chance of being sustained.

**Figure 2:**
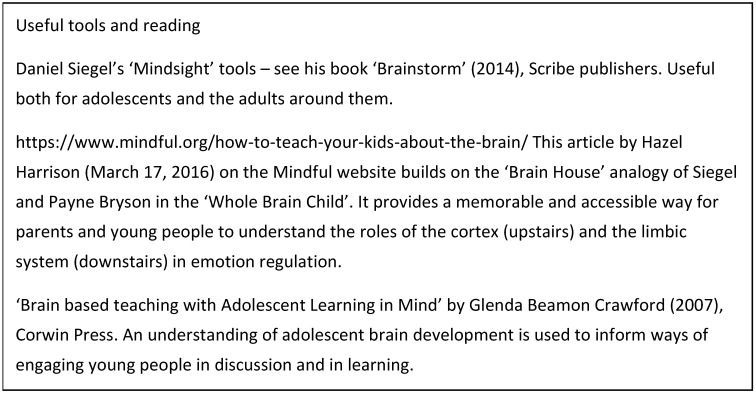
Resources that apply the knowledge.

Understanding the challenges and opportunities of adolescence may help us to engage with young people. Supporting them in understanding the connections between what is going on in their brains, and in their thinking, feelings and actions may help them towards a greater sense of mastery (see Figure 2).

### 3.2. Indications for Future Research

Research focused on how to help in a way that young people find helpful is important. We can reduce barriers to discussing topics such as body image by understanding how young people would prefer to talk about it.

Many studies find heterogeneity within illness groups. Pursuing research across multiple illness groups which focuses on illness-related variables such as visibility of the condition, duration of illness, and impact on lifestyle may be fruitful.

Cognitive biases stabilise in adolescence and play a role in how they manage certain challenges [31]. For example, we know that a cognitive bias towards interpreting pain as a ‘high threat’ situation is predictive of continuing pain. Those who see it as low threat tend to continue with activities as much as possible and are more likely to recover [32]. Further research into this may help us to intervene while the brain is still more plastic and amenable to change.

## 4. Conclusions

Adolescence is a critical period for brain development. Brain changes enable young people to venture out into the world, learn about their limits and form social identities separate from their families, paving the way for independence. Young people who are living with a chronic health condition face additional challenges in achieving these tasks of adolescence. Understanding their context may help us to ease their way.
